# Iron Dextran Increases Hepatic Oxidative Stress and Alters Expression of Genes Related to Lipid Metabolism Contributing to Hyperlipidaemia in Murine Model

**DOI:** 10.1155/2015/272617

**Published:** 2015-01-18

**Authors:** Maísa Silva, Joyce Ferreira da Costa Guerra, Ana Flávia Santos Sampaio, Wanderson Geraldo de Lima, Marcelo Eustáquio Silva, Maria Lucia Pedrosa

**Affiliations:** ^1^Basic Department-Health Area, Federal University of Juiz de Fora, Campus Universitário s/n, 36036-900 Juiz de Fora, MG, Brazil; ^2^Research Center in Biological Sciences (NUPEB), Federal University of Ouro Preto, Campus Morro do Cruzeiro s/n, 35400-000 Ouro Preto, MG, Brazil; ^3^Program of Health and Nutrition, Federal University of Ouro Preto, Campus Morro do Cruzeiro s/n, 35400-000 Ouro Preto, MG, Brazil; ^4^Department of Biological Sciences, Federal University of Ouro Preto, Campus Morro do Cruzeiro s/n, 35400-000 Ouro Preto, MG, Brazil; ^5^Department of Foods, Federal University of Ouro Preto, Campus Morro do Cruzeiro s/n, 35400-000 Ouro Preto, MG, Brazil

## Abstract

The objective of this study was to investigate the effects of iron dextran on lipid metabolism and to determine the involvement of oxidative stress. Fischer rats were divided into two groups: the standard group (S), which was fed the AIN-93M diet, and the standard plus iron group (SI), which was fed the same diet but also received iron dextran injections. Serum cholesterol and triacylglycerol levels were higher in the SI group than in the S group. Iron dextran was associated with decreased mRNA levels of *pparα*, and its downstream gene *cpt1a*, which is involved in lipid oxidation. Iron dextran also increased mRNA levels of *apoB-100*, *MTP*, and *L-FABP* indicating alterations in lipid secretion. Carbonyl protein and TBARS were consistently higher in the liver of the iron-treated rats. Moreover, a significant positive correlation was found between oxidative stress products, *lfabp* expression, and iron stores. In addition, a negative correlation was found between *pparα* expression, TBARS, carbonyl protein, and iron stores. In conclusion, our results suggest that the increase observed in the transport of lipids in the bloodstream and the decreased fatty acid oxidation in rats, which was promoted by iron dextran, might be attributed to increased oxidative stress.

## 1. Introduction

Iron is a first-line prooxidant that modulates the clinical manifestation of various systemic diseases, including diabetes, steatosis, and subclinical inflammation, which has been recognized as the dysmetabolic iron overload syndrome (DIOS) [1]. This effect may result from the ability of free iron to catalyze the formation of highly reactive radicals, such as the hydroxyl radical from superoxide and hydrogen peroxide [2]. The generation of free radicals might be involved in dyslipidemia and consequently in the pathogenesis of hepatic steatosis [3] and cardiovascular disease [4]. Previous data suggests a link between lipids and iron metabolism and that this relationship is influenced by oxidative stress [5–7]. However, despite a considerable amount of data indicating an increase of both O_2_^−^ and iron content in tissue leads to increased OH^−^ formation, the mechanism of involvement and the role of iron in early stage dyslipidemia remain to be elucidated.

In the liver, a complex network of nuclear receptors coordinately regulates the expression of enzymes involved in different steps of lipid metabolism, from fatty acid (FA) oxidation and uptake to triacylglycerol (TAG) synthesis, accumulation, and/or secretion [8]. Sterol regulatory element-binding proteins (SREBPs) are a family of transcription factors that regulate lipid homeostasis by controlling the expression of a range of enzymes required for endogenous cholesterol, fatty acid, TAG, and phospholipid synthesis. Cholesterol biosynthesis is partially governed at the transcriptional level by* srebp-2* [9], which preferentially activates many of the genes in the cholesterol biosynthesis pathway [10].* srebp-2* can regulate cholesterol homeostasis via its ability to bind and activate the promoters of genes encoding LDL receptor (LDL-R) and HMG-CoA reductase (HMG CoA-R).

Peroxisome proliferator-activated receptor *α* (*pparα*) is a transcription factor involved in liver FA degradation. It is expressed in the liver where it accelerates the transcription of proteins and enzymes for FA catabolism. It plays an important role in the metabolic homeostasis of FAs through the regulation of target genes encoding enzymes for FA oxidation and transport, such as carnitine palmitoyltransferase 1 (CPT1), which controls mitochondrial *β*-oxidation. A growing body of evidence supports a role for *pparα* in the development of liver disease, and disabling the* pparα* gene is known to increase hepatic triglyceride accumulation, especially under fasting conditions [11, 12]. In addition, an increase in oxidative stress can also cause changes in the expression of* pparα* [13].

Triglyceride release as very low-density lipoprotein (VLDL) requires the coordinated functions of apolipoprotein B-100 (apoB-100) and microsomal triglyceride transfer protein (MTP) [14]. ApoB-100 is an essential protein required for the assembly and secretion of VLDL from the liver [15]. MTP acts as both a lipid transfer protein [16] and a facilitator of apoB folding and translocation [17, 18]. MTP facilitates the transfer of four major lipid classes (free cholesterol, phospholipids, triglycerides, and cholesterol esters) to the nascent apoB-containing lipoprotein particle [19]. In addition, liver fatty acid binding protein (L-FABP), which is highly abundant in the cytosol of liver parenchymal cells, facilitates FA transport and utilization [20]. Variations in hepatic expression levels of both L-FABP [21] and MTP [22] control the flux of FAs into glycerolipid biosynthesis and VLDL assembly and secretion. However, the expression of these proteins can be altered with an increase in oxidative stress, resulting in dyslipidemia [23, 24].

To further investigate the hyperlipidemic effect of iron dextran and the possible underlying mechanism, we first focused on its effects on serum cholesterol and triacylglycerol levels. To identify the mechanisms of the hyperlipidemic effects at the molecular level, we determined mRNA expression of genes involved in lipid oxidation and triglyceride and cholesterol release from the liver and the association with enhanced oxidative stress.

## 2. Materials and Methods

### 2.1. Animals

Eight-week-old male Fischer rats (*n* = 14), weighing approximately 180 g, were obtained from the School of Nutrition, Federal University of Ouro Preto, MG, Brazil. Animals were individually housed in wire-bottomed metabolic cages and kept in a room with controlled conditions and food and water provided* ad libitum*. The standard group (S) was fed a standard diet (AIN-93M). The standard plus iron group (SI) was fed the standard diet and was given intraperitoneal injections of 100 g/L iron dextran (Sigma, St. Louis, MO, USA). For all experiments, injections were given at a total dose of 40 mg, divided into 10 mg/d dose, according the protocol by Miron et al. [25], S group received saline solution. On experimental day 28, fasting rats of 12 h were anesthetized and euthanized by total brachial plexus bleeding. Blood samples were collected and centrifuged at 10,000 ×g for the measurement of serum components. The liver was removed, weighed, and stored in liquid nitrogen or buffered formaldehyde for subsequent biochemical and histopathological analysis, respectively. All procedures were approved by the UFOP Ethics Committee on Animal Use (CEUA; OF 034/2008).

### 2.2. Assay Methods

The liver concentration of nonheme iron was analyzed after digestion in 2 mL of acid solution (3 mol/L HCl and 10% trichloroacetic acid) for 20 h at 65°C. Digested samples were mixed with chromogen reagent containing orthophenanthroline [26]. Serum triacylglycerol and cholesterol levels were measured enzymatically using Labtest kits 59—4/50 and 60—2/100, respectively (Lagoa Santa, MG, Brazil). After the precipitation of low-density lipoprotein cholesterol (LDL-C) and VLDL cholesterol with phosphotungstic acid/MgCl_2_, high-density lipoprotein cholesterol (HDL-C) level was determined from the supernatant using Labtest kit number 13. The level of other cholesterol fractions was calculated as the difference between the levels of total and HDL cholesterol. In addition, Labtest kits 53, 42, and 84 were used to measure the activities of serum alanine aminotransferase (ALT), aspartate aminotransferase (AST), and glucose concentration, respectively.

### 2.3. Lipid Peroxidation and Protein Oxidation Analysis

Liver samples from all rats were homogenized in 0.1 M Tris-HCl buffer (pH 7.4). Peroxy lipid content was measured according to Buege and Aust [27] and was calculated using the molar extinction coefficient of the complex TBA-MDA (1.56 × 105 L/mol/cm). Values are expressed in nmol/mg of protein. Carbonyl protein was measured using a modified version of the method by Levine et al. [28]. The carbonyl content was calculated based on the molar extinction coefficient of DNPH (22,000 L/mol/cm) and expressed as nmol/mg of protein. The total liver protein content was determined according to Lowry et al. [29].

### 2.4. Histopathological Evaluation

Liver fragments not exceeding 4 mm in diameter were fixed in 10% formaldehyde solution and then dehydrated, diaphanized, and embedded in paraffin. Paraffin sections of approximately 4 *μ*m were obtained by sectioning embedded fragments on a rotary microtome. Sections were mounted on glass slides previously cleaned and degreased. Slides were stained with hematoxylin and eosin for the visualization of histological changes and with Perls' technique for determination of tissue iron pools.

### 2.5. Quantitative Real Time PCR

TRIzol RNA extraction was completed using the RNAgents Promega-Total RNA Isolation System (Madison, USA) according to the manufacturer's recommendations. Total RNA concentration and purity were determined by spectrophotometric analysis at 260 and 280 nm on a NanoVue spectrophotometer (GE Healthcare, United Kingdom). cDNA was synthesized from total RNA using GeneAmp RNA PCR (Applied Biosystems, Foster City, USA). Briefly, cDNA was prepared in a 20 *μ*L reaction using MultiScribe (50 U/*μ*L) Reverse Transcriptase and Oligo d(T)_16_ primers (Applied Biosystems, Foster City, USA). Gene expression was analyzed using SYBR Green PCR Master Mix (Applied Biosystems). The primers used to amplify* pparα*,* srebp-2*, HMG CoA reductase, the LDL receptor, and the reference gene 18S have been previously described by Li et al. [30], Xiong et al. [31], and Reena et al. [32]. The primer sequences for apoB-100 (forward primer 5′-AGTAGTGGTGCGTCTTGGATCCA-3′ and reverse primer 5′-ACTCTGCAGCAAGCTGTTGAATGT-3′) were derived from the* Rattus norvegicus* genome (National Center for Biotechnology Information GenBank, accession number NM_019287) and were constructed using the Primer-BLAST Program (http://www.ncbi.nlm.nih.gov/tools/primer-blast/). The primer sequence for MTTP (forward primer 5′-CGACGGTGACGATGATCAACT-3′ and reverse primer 5′-TGACCCGCATTTTCGACATT-3′) was also derived from the* R. norvegicus* genome (National Center for Biotechnology Information GenBank, accession number NM_001107727.1). The primer sequence for L-FABP (forward primer 5′-CATCCAGAAAGGGAAGGACA-3′ and reverse primer 5′-CACGGACTTTATGCCTTTGAA-3′) was derived from the* R. norvegicus* genome (National Center for Biotechnology Information GenBank, accession number NM_012556.2). The primer sequence for* cpt-1a* (forward primer 5′-ATATTGGGCACAGTCCCCTG-3′ and reverse primer 5′-TGTGAAGAAACAACCCCCAGA-3′) was derived from the* R. norvegicus* genome (National Center for Biotechnology Information GenBank, accession number NM_031559.2).

Primers were previously tested in conventional reverse transcription PCR, in which a single band of the expected amplicon size was obtained. Quantitative (q)PCR was performed using an ABI 7300 Real Time PCR Instrument (Applied Biosystems) for 40 cycles at 95°C for 15 sec and at 60°C for 60 sec. Melting curve analysis of amplification products was performed at the end of each PCR reaction to confirm that a single PCR product was detected. Each sample was run in triplicate, and PCR reactions without the addition of the template were used as blanks. The results were normalized to the reference gene, and fold changes in expression were calculated using threshold cycle (Ct) values.

### 2.6. Statistical Analysis

Data are expressed as the means ± standard deviation (SD). All data were analyzed using the Kolmogorov-Smirnov normality test and were normally distributed. Statistical comparisons were made using Student's *t*-test. Correlation calculations were done using the Pearson correlation coefficient. In all tests, the results were considered significant with *P* values ≤ 0.05.

## 3. Results

### 3.1. Iron Status

The parameters related to iron status were measured to evaluate the effectiveness of iron dextran treatment in increasing the hepatic iron concentration. As expected, hepatic nonheme iron concentration was increased (*P* < 0.0001) in group SI relative to group S (Table 1).

### 3.2. Effect of Iron Overload on Serum Lipid and Glucose Profiles and on Hepatic Function

As can be observed in Table 1, the serum level of total cholesterol was higher (*P* < 0.05) in group SI than in group S. The same profile was observed for the concentration of cholesterol in other fractions. HDL-C levels in group S and group SI were not significantly different. Regarding triacylglycerol concentrations, again the rats in group SI showed higher levels compared with group S, and a similar profile was observed for glucose concentration. No difference between the groups was detected in aminotransferase activities.

### 3.3. Effect of Iron Overload on Lipid Peroxidation and Protein Oxidation in the Liver

As shown in Table 1, the level of liver TBARS of SI-fed animals was 2.18-fold higher (*P* < 0.0001) than those in group S. Furthermore, a 2.13-fold increase (*P* < 0.0001) in protein oxidation caused by iron overload was seen in group SI compared with group S.

### 3.4. Histopathological Evaluation of Liver Tissue

**Figure 1:**
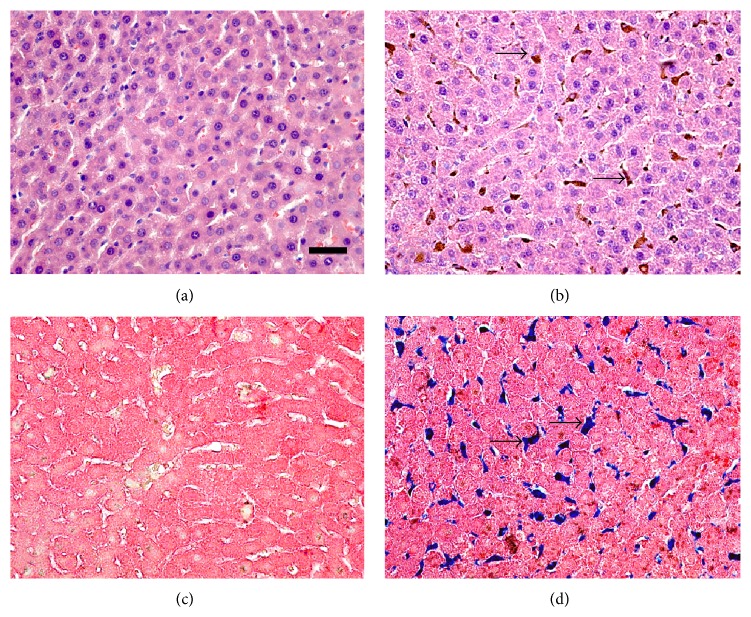
Photomicrographs of histological liver sections. (a) Standard group showing normal histology. (b) Standard group receiving the diet iron injections (group SI) featuring ballooned hepatic cells suggesting a hydropic degenerative process and focal granulomatous inflammation, cytoplasmic iron store in hepatocyte and Kupffer cell (arrow). (c) Control group showing normal histology. (d) Iron group receiving iron injections and featuring iron deposits in hepatocytes (arrow). ((a) and (b)) Hematoxylin & Eosin staining. ((c) and (d)) Perls staining. Bar = 50 *μ*M.

Histological analysis showed that, generally, the liver of animals in group S was normal (Figure 1(a)). The animals in group SI showed a moderate presence of ballooned hepatic cells, which were diffuse throughout the parenchymal tissue. This is usually associated with granulomatous inflammatory foci, and tissue iron stores were located primarily in hepatocytes and Kupffer cells (Figure 1(b)). Histological analysis with Perls' technique showed iron deposits in group SI (Figure 1(d)), whereas none were observed in group S (Figure 1(c)).

### 3.5. Effect of Iron Overload on Hepatic Gene Expression

**Figure 2:**
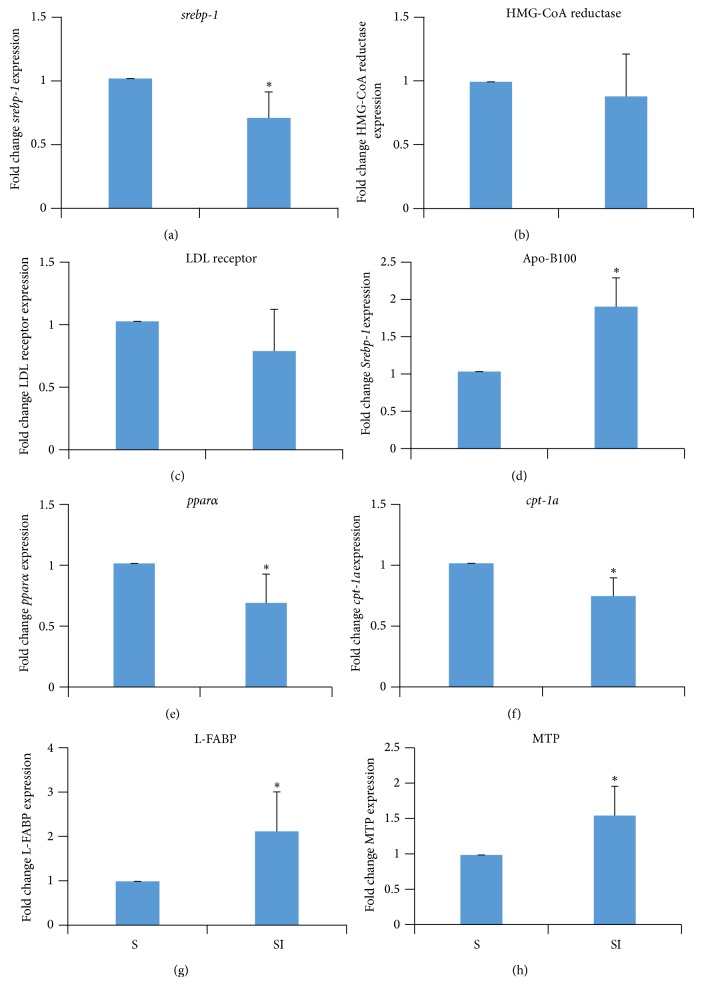
Levels of gene expression in the liver of rats offered a standard diet (S) and rats that received injections of iron dextran (SI). Values are the mean ± standard deviation (*n* = 7). (a) Levels of relative* srebp-2* (sterol regulatory element-binding proteins-2)/18S expression. (b) Levels of relative HMG-CoA reductase/18S expression. (c) Levels of relative LDL receptor/18S expression. (d) Levels of relative ApoB100 (apolipoprotein B-100)/18S expression. (e) Levels of relative PPAR alpha (peroxisome proliferator-activated receptor alpha)/18S expression. (f) Levels of relative* cpt1a* (carnitine palmitoyltransferase 1)/18S expression. (g) Levels of relative L-FABP (liver fatty acid binding protein)/18S expression. (h) Levels of relative MTP (microsomal triglyceride transfer protein)/18S expression. ^*^ *P* < 0.05 with respect to the S group (student's* t*-test).

Liver expression of *srebp-2* was lower (*P* > 0.01) in group SI than in group S (Figure 2(a)). There was no statistically significant difference between groups with respect to the expression levels of HMG-CoA reductase and the LDL receptor in the liver (Figures 2(b) and 2(c)). However, liver expression of* apoB100* was higher (*P* > 0.01) in group SI than in group S (Figure 2(d)). The expression of *pparα* and *cpt1a* was lower in the SI than in the S group (*P* > 0.05 and *P* > 0.01, resp., Figures 2(e) and 2(f)), and expression of* L-FABP* and* MTP* was higher in the SI than in the S group (*P* > 0.05 for both, Figures 2(g) and 2(h)).

### 3.6. Correlation Analysis

**Figure 3:**
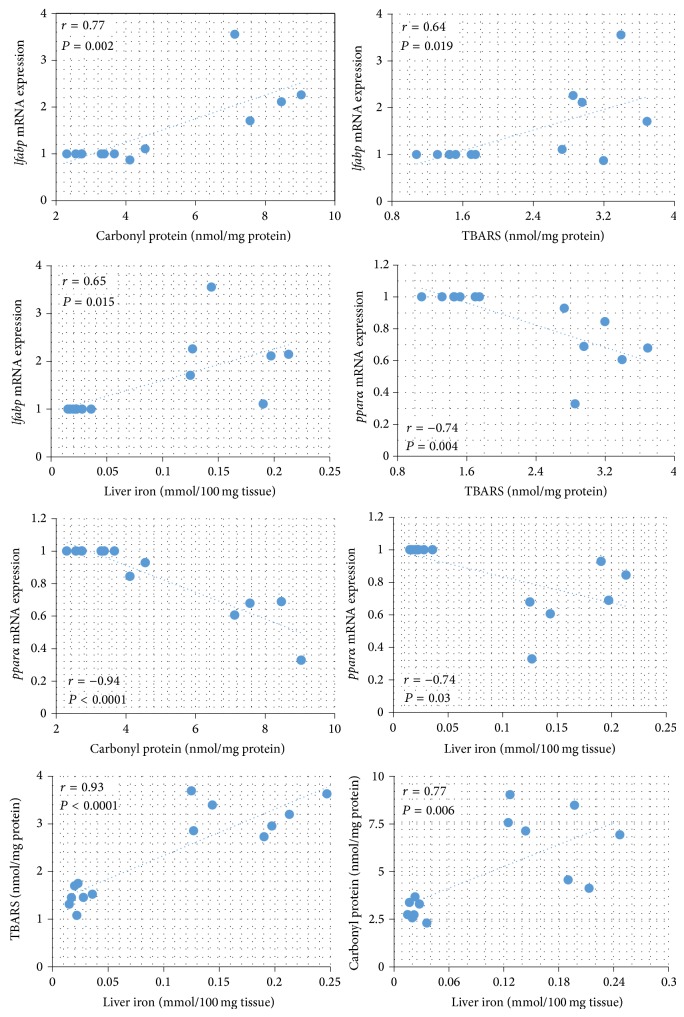
Correlations between levels of relative L-FABP (liver fatty acid binding protein)/18S expression and iron stores, carbonyl protein, and TBARS level, correlations between levels of relative PPAR alpha (peroxisome proliferator-activated receptor alpha)/18S expression and iron stores, carbonyl protein, and TBARS level, and correlations between carbonyl protein, TBARS level, and iron stores of experimental animals. The Pearson *r* value was calculated and a positive or negative correlation is statistically significant when *P* < 0.05.

In Figure 3, linear regression analysis of the groups showed that* lfabp* mRNA expression was positively correlated with carbonyl protein, TBARS, and iron and* pparα* mRNA expression was negatively correlated with carbonyl protein, TBARS, and iron stores. There was a significant association of iron stores in relation to TBARS and carbonyl protein measured in the liver. In particular, iron stores were positively correlated with TBARS and carbonyl protein.

## 4. Discussion

The present study used an* in vivo* rat model to demonstrate that iron dextran can promote hypercholesterolemia and hypertriglyceridemia. These were associated with downregulation of genes involved in FA *β*-oxidation and upregulation of genes involved in the transport of lipids in the liver. Our data suggests that oxidative stress has a causal role.

The hepatic iron levels observed in the present study were moderate compared to patients with symptomatic hemochromatosis, who exhibit a 16- to 30-fold increase in these levels [33]. This was also below the critical threshold (>22-fold increase) in which hepatocellular injury has been observed [34]. Association between excessive systemic iron and diabetes emerged by the observation that the incidence of diabetes is increased in classical hereditary hemochromatosis [35]. Epidemiological data show a positive correlation between body iron pools and development of glucose intolerance seen in diabetes type 2 [36, 37]. Our experimental model also might be used to study this association.

*pparα* has an important role in the metabolic homeostasis of FAs through the regulation of target genes encoding enzymes for FA oxidation and FA transporters [11, 12].* pparα* activation promotes FA oxidation, ketone body synthesis, and glucose sparing. Disabling the* pparα* gene is known to increase hepatic triglyceride accumulation, especially under fasting conditions [38–40]. Pharmacological activation of* pparα* has been shown to lower hepatic triglyceride levels and to effectively attenuate steatohepatitis [12, 39, 41]. Furthermore, hypercholesterolemia can decrease protein expression of PPARs in the rat liver [42], and the hyperlipidemic fatty liver rat model also exhibits a decrease in this protein [43]. Iron dextran decreased* pparα* expression, suggesting that it would decrease FA oxidation, thus increasing serum triacylglycerol concentration. Bonomo et al. [7] showed similar effect of iron on the expression of PPAR alpha in hypercholesterolemic hamsters; however this study did not evaluate triacylglycerol metabolism. Oxidative stress and inflammation modulate PPAR receptors in diabetes [13], suggesting that increased oxidative stress promoted by iron dextran may have resulted in the decreased* pparα* expression, this was also correlated in this study. The oxidative stress could alter mRNA expressions involved lipid metabolism because reactive oxygen species can alter transcription factors, inducing or repressing the expression of genes. We suggest that reactive oxygen species can affect the translocation of transcription factors sensitive to the redox state to the nucleus. Changes in the mRNA expressions involved lipid metabolism may be due to the oxidation of transcription factors; NF-E2-related factor 1 (Nrf1) was recently found as regulator of hepatic lipid metabolism [44]. We found that liver *cpt-1a* expression was markedly decreased in iron dextran, which is in agreement with previous studies by Koonen et al. [45] and Lelliott et al. [46], who observed the same effect in dyslipidemic conditions. These results indicate that the increase in serum triacylglycerol may be the result of decreased lipid oxidation and that the iron dextran could decrease FA oxidation by the inhibition of* pparα* and its target genes in the liver.

ApoB-100 is associated with hepatic-derived non-HDL-C and is incorporated into nascent lipoprotein particles, along with cholesterol and triglycerides [47]. The increased mRNA levels of* apoB-100*, which were promoted by iron dextran, suggest that an increase in the overall secretion of VLDL is caused by modifications in the packaging of this lipoprotein. In addition to the upregulatory effect on* apoB-100*, supplementation of iron increased the expression of* MTP* in this study. MTP plays an important role in VLDL assembly by mediating the transfer of hepatic lipids to nascent apoB [27]. It has been reported that* MTP *gene expression is enhanced in the liver of both fructose-fed hamsters and obese diabetic mice [48]. In the current study, *MTP* mRNA expression was significantly increased in iron overloaded rats, which may contribute to increased plasma cholesterol and triacylglycerols, as MTP inhibitors have been developed and shown to be effective lipid-lowering drugs in animal models [49]. Studies with antioxidant compounds show that they are able to reverse the increase in MTP and apoB-100 that is seen in lipid rich diets [24, 50]. Therefore, iron dextran can increase oxidative stress and this can alter the expression of these proteins.

L-FABP is one of the most abundant proteins in the liver, accounting for 2-3% of the total cytosolic protein pool [51]. As such, it can be expected to play an important role in cellular homeostasis. It serves as an intracellular transporter of lipophilic ligands, such as long-chain FAs [52]. Its expression is modulated by developmental, hormonal, dietary, and pharmacological factors, and it may change in response to specific diseases such as cholestasis [53] or alcohol-induced liver injury [54]. Previous studies have demonstrated that, in fatty liver rats, the expression of* L-FABP* mRNA was increased in comparison to the control group [43]. Thus, the iron dextran promotes effects similar to those found in the consumption of a high fat diet. L-FABP may have an additional cytoprotective role as it contains one cysteine and several methionine residues [55] and forms a large portion of the intracellular protein pool. Previous studies have shown that L-FABP is an important member of the hepatocellular antioxidant defense system, reducing ROS levels during periods of oxidative stress [56]. The positive correlation between products generated by oxidative stress and L-FABP in this study also suggests that iron dextran can cause alterations in this expression via redox imbalance. Abe et al. [23] showed that rosuvastatin administration reduces L-FABP level, in part due to reducing oxidative stress, thus strengthening our hypothesis. However, our study is limited in that only the gene profile was analyzed; thus, it is important to confirm if alterations of genes expression are reflected by protein levels.

The mRNA level of the other factors involved in FA metabolism (including SREBPs) was significantly increased in the livers of mice under conditions of elevated oxidative stress [57]. Furthermore, an upregulation of* srebp-2* stimulates hypercholesterolemia and hepatic steatosis because* srebp-2* is activated in response to the reduction in hepatic cholesterol content. However, the alterations observed in *srebp-2*, and the genes regulated by it (HMG-CoA reductase and LDL-R) does not seem to be involved in the mechanism of action of iron promoting hypercholesterolemia. Iron-treated rats showed higher serum cholesterol levels and reduced expression of* srebp-2* (and tendency of reduced expression of HMGCR and LDLR). The reduction in* srebp-2* expression may indicate feedback regulation in response to high cholesterol levels.

## 5. Conclusions

The present study demonstrated that iron dextran increased oxidative stress, which was associated with the altered expression of genes related to lipid metabolism and therefore contributing to hyperlipidemia.

## Figures and Tables

**Table 1:** Iron status in liver, glycemic and lipids profile, oxidant/antioxidant status in liver of rats offered a standard diet (S) and rats that received injections of iron dextran (SI).

	S group	SI group	*P* values
Liver iron *µ*mol/100 mg tissue	0.02 ± 0.01	0.18 ± 0.05^*^	<0.0001
Cholesterol mmol/L of serum	1.26 ± 0.27	1.77 ± 0.33^*^	<0.05
HDL cholesterol mmol/L of serum	0.83 ± 0.17	0.93 ± 0.17	>0.05
Other fraction cholesterol mmol/L of serum	0.42 ± 0.28	0.84 ± 0.37^*^	<0.05
Triacylglycerol mmol/L of serum	1.15 ± 0.22	2.06 ± 0.59^*^	<0.01
Glucose mmol/L of serum	6.24 ± 0.96	7.34 ± 0.46^*^	<0.05
ALT (U/mL of serum)	23.72 ± 4.34	25.68 ± 7.32	>0.05
AST (U/mL of serum)	55.26 ± 4.26	56.92 ± 3.61	>0.05
Liver carbonyl protein (U/mg of protein)	2.95 ± 0.5	6.83 ± 1.86^*^	<0.0001
Liver TBARS (U/mg of protein)	1.47 ± 0.23	3.21 ± 0.38^*^	<0.0001

HDL, high-density lipoprotein;

Values are shown as the mean ± standard deviation (*n* = 8). Data were analyzed by *t*-test. Statistical differences are shown by ∗.
